# Integration of partners of young women with cancer in oncofertility evidence‐based informational resources

**DOI:** 10.1002/cam4.3377

**Published:** 2020-08-30

**Authors:** Vânia Gonçalves, Pedro L. Ferreira, Gwendolyn P. Quinn

**Affiliations:** ^1^ Centre for Health Studies and Research of the University of Coimbra (CEISUC) Faculty of Economics University of Coimbra Coimbra Portugal; ^2^ Departments of Obstetrics and Gynecology and Population Health Grossman School of Medicine New York University New York NY USA

**Keywords:** cancer, fertility, informational resources, oncofertility, partners, young women

## Abstract

Oncofertility has evolved over the years, with a prodigious amount of research documenting the importance of fertility for young patients with cancer, and the potential impact that fertility impairments due to cancer treatments has on their Quality of Life (QoL). Multiple professional bodies and scientific societies have included fertility as an integral part of clinical management. Clinical guidelines advocate that health professionals have the duty to discuss the risk of infertility and fertility preservation options as early as possible and refer to fertility specialists when appropriate. Collectively, fertility decisions are regarded as difficult for both patients and providers. Since providing fertility‐related information is vital for better decision making, researchers and policy makers have concentrated their efforts in developing educational tools to aid decisions and guidelines to optimize the delivery of this information, focusing mainly on patients‐providers and largely neglecting the role and influence that partners play in this process. Here, we reflect on the importance of partners in fertility decisions, with a focus on the provision of fertility‐related information that is also geared towards partner. We highlight the need to involve partners in fertility discussions, and that their needs should be taken into account in both clinical guidelines and in the development of educational tools, for an optimal decision‐making process.

## Graphical abstract

There is a need to involve partners in fertility discussions. Partners' needs should be taken into account in both clinical guidelines and in the development of educational tools, for an optimal decision‐making process

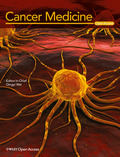

## 1 INTRODUCTION

Since the first studies were published in the nineties, fertility preservation in oncology settings has become an increasingly investigated topic in the literature, namely, in the USA and, more recently, worldwide. Over the years, the potential struggle that young patients diagnosed with cancer may face when confronted with the real possibility of losing their childbearing capabilities due to cancer treatments has been extensively documented. Despite a cancer diagnosis, many young patients dream of creating their own family or of extending their existing family in the future. The importance of fertility among young patients and the potential implications of its impairment on their quality of life (QoL) is well established. The use of assisted reproductive technology (ART) has become a hope to help men and women become parents in the future. For female patients, cryopreservation of oocytes, embryos, ovarian tissue, ovarian suppression, and transposition are available fertility preservation procedures. For men, cryopreservation of semen is the established method for fertility preservation. Adoption and third‐party reproduction are other options for patients who were not able to use fertility preservation techniques prior to the start of cancer treatments or for whom techniques were unsuccessful. The need to include fertility as an integral part of the clinical management of these young patients has been validated by several professional bodies and scientific societies by recognizing the significance of providing information and discussing potential fertility loss and offering fertility preservation to people diagnosed with cancer.^1^, ^2^, ^3^, ^4^, ^5^, ^6^, ^7^, ^8^, ^9^, ^10^, ^11^, ^12^, ^13^, ^14^, ^15^, ^16^, ^17^, ^18^, ^19^ Guidelines were developed by national and international organizations advocating that health professionals involved in young patients cancer care have the duty to inform and discuss with their patients the risk of infertility and fertility preservation options as early as possible and refer to fertility specialists when appropriate.^2^

Given the differences between female and male infertility risks, fertility preservation options, and the nature of the techniques and time required to implement them, this manuscript will focus solely on young women diagnosed with cancer.

Significant and rapid advances have been made in oncofertility, a discipline that merges oncology with fertility,^20^ in terms of effectiveness of techniques and demands from patients and health professionals to have access to it.^21^ However, there are still issues regarding fertility‐related communication and information provision that are lacking and may impact the decision‐making process. To fully optimize fertility preservation decision making, it is imperative to understand the role of partners. It is crucial that partners' perspectives are considered in the development of patient and clinician educational materials.

This narrative review addresses the importance of partners in the process of oncofertility decision making. Firstly, we provide current evidence on the importance of the partner during the decision‐making process. Next, we focus on the vital role of the inclusion of partners in fertility‐related information. This information includes the development of clinical and educational evidence‐based resources, such as decision aids and clinical practice guidelines for fertility preservation in women with cancer.

## 2 FERTILITY‐RELATED DECISIONS IN A CANCER CONTEXT—THE IMPORTANCE OF THE PARTNER

Deciding on fertility preservation options is already an emotional‐charged process^22^; however, when these decisions take place concomitantly with a cancer diagnosis and other cancer treatment decisions, they may place the patient and the medical team in a unique challenging position. It is consensual that fertility decisions in oncological settings involving young patients are often difficult for all the parties involved. Decisions about fertility preservation and associated needs must be achieved during a narrow window of time; and these decisions also involve considerations of future difficult ethical and legal issues such as the length of time to store frozen gametes, donating banked gametes to infertile couples, and whether embryos created with one partner would be accepted by another partner.^23^ Another complex future decision regards the fate of unused stored gametes in the event of death.^24^, ^25^ Many of these decisions occur at the time a decision related to cancer treatment is also made. An intricate set of factors at a personal, medical, familial, legal, and spiritual level define the ultimate decision which may have short and long‐term effects on the patient's well‐being and mental health.^26^

Research on the process of fertility‐related decision making in the context of cancer places the interaction between the patient and the health‐care professional (namely, the oncologist and the fertility specialist) at the cornerstone of the decision. However, and without taking any legitimacy of value to the key players identified above, research has consistently documented that a significant number of participants in the studies are, in fact, women who are married or in a committed relationship.^27^ Cancer‐related infertility may impact relationships, with some women expressing concerns about discussing fertility issues with their partners.^28^ Extrapolating from research findings in non‐cancer populations, since this area of research in cancer patients still needs to be further investigated, the process of fertility decision making contributes to the experience of psychological distress for couples, as being infertile is associated with marital distress and a decrease in marital and life satisfaction.^29^

Culturally, partners have a strong influence on women's decisions to become a mother and, in general, the decision to initiate or extend the family concerns both members of the partnership. Such decisions are based on a consensus between the couple about both having a child and sustained by their commitment to endure the fertility treatment together.^30^ This seems highly relevant in a cancer context. To illustrate this, our recent study showed that young women were more likely to preserve their fertility if their partners' desire to be a father increased after the cancer diagnosis.^31^ This finding corroborates previous research, which demonstrated that, among other factors, the women's acceptance of fertility preservation was related to the wishes of their partners.^32^ Furthermore, many of these young women diagnosed with cancer identified their partners/spouses as the most useful and more often used person to discuss fertility‐related information,^33^ also stressing that their partners were involved in the fertility‐related decision‐making process.^31^, ^34^ ^,35^ Supporting this view, another study showed that women expressed that the ideal setting for fertility preservation discussions to occur were in the presence of their partner before the cancer treatment started.^36^ Similarly, health professionals involved in cancer care also acknowledged that fertility is an important concern not only for young patients but also for their partners.^37^ Among noncancer populations, research has advocated that for infertility patients, clinicians should encourage the active participation of both partners in fertility discussions and decisions.^38^ This practice is substantiated by the European Society of Human Reproduction and Embryology (ESHRE) guidelines, developed for fertility information provision for patients in the general community on psychosocial care in infertility treatment, which recommends the active involvement of partners in fertility decision making.^39^ In parallel, NICE (National Institute for Health and Care Excellence) guidelines for fertility problems recommends that couples who experience problems in conceiving should be seen together because both partners are affected by decisions surrounding investigation and treatment.^6^ Findings from oncofertility research also support this inclusion of partners in fertility discussions for cancer populations.^31^, ^40^

## 3 FERTILITY DECISIONS—VITAL ROLE OF INFORMATION PROVISION

A vital component of any decision‐making process in medical settings is the provision of patient information that is relevant to their individual needs.^41^ Particularly, to reach a decision about using fertility preservation, patients need to be fully informed about potential fertility risks and the fertility options being offered to them,^42^ understand the values that affect decision making and adapt to new information.^43^ Research suggests women benefit from, and have reduced remorse and regret about their fertility decisions, when provided opportunities to learn about potential infertility prior to treatment. This remains true even among women for whom no fertility preservation options were available or elected.^10^, ^44^ Lack of information leads to greater decisional conflict,^22^ which is the state of uncertainty about the course of an action to be taken. This tends to be associated with emotional distress, future regret or/and blame, and delayed decision making.^32^ Despite the rapid advances in oncofertility, recent papers still report that women have expressed absence of fertility discussions prior to cancer treatment, and have indicated that their needs regarding fertility information are not always met by their health‐care providers.^31^, ^45^, ^47^ Young patients consider the provision of fertility‐related information a priority.^48^ Beyond the conventional patient‐provider discussions, patients also value educational tools, such as written materials, to inform their choices about fertility preservation.^49^ Particular interest has been seen in the development and validation of Decision Aids (DAs) aimed to ease the path to reach fertility decisions that are adequately optimized and tailored to patients' needs.^46^

Beyond the possible emotional support that partners may provide to patients during the decision‐making process, for example, by accompanying them to the consultations; it is relevant to assume that partners are also active beneficiaries of the all processes, with justifiable informational needs in order to contribute to an informed fertility decision. For example, a couple may need to deal with the legal complexities involving some fertility decisions which may impact each partner, or have to adapt to a new reality as third‐party reproduction becomes the only available option. One might therefore also expect to see partners taken into account as key stakeholders, beneficiaries, and players during the fertility decision‐making process acknowledged in the development of fertility‐related informational tools and guidelines. It is worth examining partners’ acknowledgement in the development of these clinical and educational evidence‐based resources, designed to educate health professionals about the need to include fertility discussions into clinical management and aid patients in optimal idiosyncratic fertility decisions, respectively.

### 3.1 Decision aids

Decision Aids (DAs) are educational materials designed to assist with treatment decision making by addressing individual values and preferences,^48^ and are particularly helpful in situations when there is limited time to make the decision.^50^ DAs help make the decision explicit, describe options available, and assist patients understanding of options as well as their possible benefits and harms. DAs assist patients in considering the options from a personal perspective, allowing them to participate with their health provider in shared decision making.^51^ A recent systematic review concluded that fertility‐related DAs for cancer patients can be effective complements to current fertility care by increasing fertility information satisfaction and knowledge and may lower decisional conflict and regret; thus, helping patents to make better‐informed decisions.^46^ The number of available written materials and online resources about fertility preservation for young cancer patients from different organizations are growing.^52^, ^53^ However, validated educational tools to support fertility decision making are still scarce,^54^ and with respect to evaluated DA include one for young women with breast cancer in English^48^ and another DA in Dutch^52^ and one DA in German for young female cancer patients.^55^

One of the first fertility‐related DAs for young breast cancer patients was developed and validated by Peate in Australia.^48^ This DA's evaluation took into consideration partner's input on the DA and collected information on their involvement during the evaluation process to assess DA's efficacy, such as whether the DA was shared with the partner, the extent to which partners had read the materials, whether the materials stimulated discussion between partners, how useful the partners considered the booklet (DA), and whether the partner contributed to the decision‐making process about fertility‐related issues. This evaluation reported that the majority of participants’ partners involved in fertility decision making.

### 3.2 Clinical practice guidelines for fertility preservation in women with cancer

Clinical practice guidelines are intended to guide health‐care professionals and patient decisions regarding appropriate, safe, and cost‐effective fertility care for women who desire biological children after a cancer diagnosis,^56^ describing available fertility preservation techniques and determine their appropriateness.^18^ Although there are inconsistencies and variability in fertility preservation recommendations among different guidelines^57^; collectively, these guidelines acknowledge fertility care as an important component of cancer management for young patients and the need for fertility discussions between health‐care professionals and the young patient before cancer treatment begins. Despite that fertility preservation field is rapidly evolving, the importance and need of involving patients' partners (if, they exist) in fertility discussions remains a neglected point in the great majority of fertility guidelines or guidelines updates in these group of patients. There are, however, few exceptions, which deserve to be acknowledged. The European Society of Breast Cancer Specialists (EUSOMA) recommends that fertility discussions should occur before the start of any cancer therapy, allowing for appropriate time for reflection and should possibly involve the partner. The Fertility Preservation Network (FertiPROTEKT), an European society of physicians and biologists specialized in fertility preservation, offers a general recommendation for including the individual wishes of the patient and their partners.^1^ The Ethics Committee of the American Society for Reproductive Medicine recommends that when a partner exists, he or she may be included in fertility discussions, adding that it is also advisable to discuss these issues with the patient individually.^13^ The clinical guidelines developed by the Clinical Oncology Society of Australia (COSA) for fertility preservation for adolescents and young adults diagnosed with cancer stress that all patients who require treatment that could compromise future fertility must be given the opportunity to discuss the effects of the treatment and available options to protect or preserve fertility with their oncologist and/or a fertility specialist and whenever appropriate discussions should include partners and families.^8^

## 4 CONCLUSION

Insights from cancer research allow us to discern that during the process of fertility discussions, which may include the use of educational resources, partners seem to play a valuable and influential role during the fertility decision‐making process. This reflects the uniqueness of fertility decisions, which contrary to many other shared medical decisions, such as treatment for a chronic condition, are characterized by a distinctive complex triadic interaction between the health‐care professional, the women, and their partner.^43^ With the knowledge that improving communication of information is essential and beneficial for better decision making and ultimately improved mental health,^58^ researchers and policy makers have concentrated their efforts in developing evidence‐based tools and guidelines to optimize the delivery of this information, focusing mainly on patients‐providers and largely neglecting the role that partners play in this process. Presently, the majority of guidelines fail to account for the importance of including partners in fertility discussions, failing to provide effective strategies or guidance to promote that inclusion. In addition, current decision validated tools offer limited space for partners input. Since partners seem to play a key role in the process of fertility decision making, we strongly suggest that future research should focus further on the partners' role and informational needs in oncofertility in order to aid clinical guidelines in the provision of a framework of specific practices that promote and improve communication among the couple (when a partner exists) and the health‐care professional. This will increase the quality of fertility discussions and support clinicians and other health professionals in their daily practice. In addition, decision tools should explore and incorporate young cancer patients’ partners' perspectives and needs. Furthermore, it should also be noted that, even when the relationship between providers and patients/partners places the latter in the center of care, the use of DAs may be very useful to lessen the existing natural knowledge asymmetry between the provider and patients/partners, which is vital for a shared decision‐making process.

## CONFLICT OF INTEREST

The authors made no disclosures.

## AUTHOR CONTRIBUTIONS

VG conceived the idea, conducted the literature search, and wrote the original and revised versions of the report. PF participated in manuscript revisions. GQ conceived the idea and participated in manuscript planning and revisions. All authors approved the final version of the manuscript.

## Data Availability

Data sharing not applicable to this article as no datasets were generated or analyzed during the current study.
